# Informal Caregiving for Individuals with Dementia and Concurrent Sensory Impairment: A Systematic Review

**DOI:** 10.1093/geroni/igaf122.1623

**Published:** 2025-12-31

**Authors:** Shaoqing Ge, Katie Trainum, Yaolin Pei, Wonshik Chee, Yuting Song, Bo Xie

**Affiliations:** University of Texas at Austin School of Nursing, Austin, Texas, United States; University of Pennsylvania, Philadelphia, Pennsylvania, United States; The University of Texas at Austin, Austin, Texas, United States; University of Texas at Austin, Austin, Texas, United States; Qingdao University, Qingdao, Shandong, China (People’s Republic); The University of Texas at Austin, Austin, Texas, United States

## Abstract

Caregivers of individuals with dementia often face the dual challenge of managing dementia alongside sensory impairments (hearing and/or vision loss). Despite the significant burden, this issue remains underexplored, with existing research largely overlooking the impact of sensory impairments on caregivers’ experiences. This systematic review examines the challenges and needs of caregivers managing both conditions. A comprehensive literature search across three databases identified 12 studies published before May 2024 in five countries. The review revealed that caregivers face increased challenges when managing both dementia and sensory impairments. Unmet needs, particularly in accessing tailored support and assistive technologies, were prevalent. Caregivers reported difficulties in communication, increased stress, and a higher risk of burnout due to the compounded effects of managing both cognitive and sensory impairments. The review highlights the importance of multidisciplinary care and interventions to address both cognitive and sensory needs. Effective strategies include integrating sensory assessments into dementia care plans, providing training for caregivers on managing sensory impairments, and ensuring access to affordable assistive technologies. Additionally, caregivers expressed a need for more comprehensive support systems, including respite care and mental health services, to alleviate the emotional and physical strain of caregiving. Enhanced caregiver education on the use of assistive devices and techniques to improve communication with sensory-impaired dementia patients is essential. Addressing these disparities is critical for providing effective care and improving the quality of life for both caregivers and sensory-impaired dementia patients. This review underscores the urgent need for targeted interventions and policies to support this vulnerable population.

